# Analysis of the prevalence of musculoskeletal disorders and occupational stress in professors of a higher education institution in the state of Pernambuco

**DOI:** 10.47626/1679-4435-2020-542

**Published:** 2021-02-11

**Authors:** Thais Elvira Nogueira Almeida, Rosa Elita de Andrade Ferreira, Luciana Ângelo Bezerra, Taciane Machado de Melo Pereira

**Affiliations:** Fisioterapia, Faculdade de Integração do Sertão - Serra Talhada (PE), Brazil

**Keywords:** RSI/WMSD, burn-out, professor, posture, occupational physical therapy

## Abstract

**Introduction:**

The consequences of technological innovations and current ways of work organization have prompted the appearance of various health conditions, namely repetitive strain disorders, work-related musculoskeletal disorders, and burn-out; these can emotionally and physically overload workers.

**Objectives:**

To evaluate the prevalence of musculoskeletal disorders and occupational stress in professors of the Serra Talhada campus of Instituto Federal do Sertão Pernambucano.

**Methods:**

Participants signed a free and informed consent form and answered to the Nordic Musculoskeletal Questionnaire. Subsequently, we applied the Maslach Burnout Inventory-Human Services Survey and evaluated the participants’ postures during teaching by using the Rapid Upper Limb Assessment tool (Ergolândia software).

**Results:**

We observed that 82% of the participants presented musculoskeletal symptoms, mainly in the lower back and lower limbs. Professors presented high scores for the burn-out syndrome, and posture evaluation indicated action levels of 3-4 (suggesting immediate intervention).

**Conclusions:**

The implementation of an occupational physical therapist in this higher education institution is recommended in order to promote ergonomic adaptations and to elaborate a protocol for workplace physical activity, ultimately aiming to prevent musculoskeletal disorders and occupational stress.

## INTRODUCTION

Technological innovation and current ways of work organization have brought upon workers various health problems, as well as emotional and musculoskeletal overloads as the main causes of occupational diseases, which are currently considered one of the greatest public health problems worldwide^1,2^. Among work-related diseases, repetitive strain injury (RSI) and work-related musculoskeletal disorders (WMSD) are noteworthy and cover various diseases and alterations that affect muscles, fasciae, blood vessels, tendons, ligaments, nerves, and joints. These are characterized by chronic and localized pain, mainly in the spine and upper limbs, resulting from work activities that mainly include repetitive movements and muscular effort^3,4^.

Professors and teachers are prone to work-related musculoskeletal pain mainly due to orthostatism when teaching classes. This position causes overloading of the spine, muscle fatigue, and static muscle loading of stabilizer muscles of the back and limbs. Prolonged standing associated to poor posture can also affect body alignment and ultimately lead to work-related diseases^5-7^.

Another type of occupational phenomenon, according to the World Health Organization (WHO)^8^, is the burn-out syndrome; it has recently been included in the International Classification of Diseases (ICD-11). The burn-out syndrome results from chronic workplace stress; it is characterized by the triad emotional exhaustion (EE), depersonalization (DP), and reduced feelings of self-realization (SR). Symptoms include feelings of lack of energy and negativism related to the job, as well as reduced professional efficacy.

The presence of a physical therapist in the workplace is necessary for promoting changes that could reduce possible risks of work-related accidents and health problems, thus resulting in increased productivity. This professional should investigate the pain and discomfort presented by employees, also reducing absenteeism^9^. Work-related muscular effort can generate poor posture habits that require an adequate process involving education and postural control for their resolution. Professors are exposed to high cognitive demands and perform activities in a dynamic and repetitive fashion. In view of these aspects, this study aimed to evaluate the prevalence of musculoskeletal disorders and occupational stress during professorship in a higher education institute in the inland of the state of Pernambuco.

## METHODS

This is a cross-sectional study performed at the Serra Talhada campus of Instituto Federal do Sertão Pernambucano (IF Sertão-PE) between June and August 2019. Our sample consisted of faculty members who met the inclusion criteria: male or female, aged 18 years and older, employed by the institution, and working at the moment of the study. Those who also worked in another company, presented neurologic sequelae, had congenital physical anomalies and/or had been subjected to amputations, as well as those who were pregnant at the moment of the evaluation were excluded from the study. The institute had 40 professors, of which 1 was excluded due to being on maternity leave, 5 were on leave, and 17 could not be located at the moment the evaluations took place. After applying the defined criteria, 17 participants constituted our final sample. This study was approved by the Research Ethics Committee of Sociedade de Ensino Superior de Serra Talhada (SESST), decision number 3 345 623, and respected the rules and guidelines for research with human beings (466/12 and 510/2016).

Participants were informed of the objectives of the study and signed a free and informed consent form before the beginning of the study. Data collection initially used a sociodemographic questionnaire specially elaborated by the researchers and containing the following variables: sex, age, duration of employment, daily workload, work-related discomfort, and physical activity. Subsequently, we used the Nordic Musculoskeletal Questionnaire (NMQ) for evaluating musculoskeletal symptoms in a standardized fashion. This is one of the main instruments used for analyzing musculoskeletal symptoms in the contexts of occupational health and ergonomics, allowing the comparison of results of epidemiological studies on this subject. In this study, we used the Brazilian version of the NMQ, validated by Pinheiro et al.^10^ in 2002^11^.

The next questionnaire used in this study was the Maslach Burnout Inventory-Human Services Survey (MBI-HSS)^12^, in its Portuguese version as published by Semedo^13^. This questionnaire contains 22 items that cover work-related feelings, distributed along 3 scales: EE, with 9 items; DP, with 5 items; and SR, with 8 items. The answers measure the frequency with which the participant experiences each feeling in an ordinal scale ranging from never (1) to every day (7). Regarding the EE scale, the level of burn-out is considered high when the participant scores higher than 27, it is considered low/moderate when the score is between 19 and 26, and low when it is lower than 19. In the DP scale, scores over 10 are considered high, while scores 6-9 are moderate and scores of less than 6 indicate a low level of burn-out. Finally, the SR scale works in an opposite direction: Scores of 40 or higher indicate a low level of burn-out, while scores of 34-39 are moderate and those lower than 33 represent a high level of burn-out.

Finally, we used the Rapid Upper Limb Assessment (RULA) method, developed by McAtamney & Corlett^14^ in 1993, for quantitatively evaluating postural, dynamic, and static risks, including strength and repetitiveness, and qualifying limb positions. The RULA method used in this work was made available through the Ergolândia 5.0 software (FBF Sistemas)^15^ for helping professionals and companies in the field of occupational health. Scores for each item are classified into action levels: level 1 (acceptable posture as long as it is not repeated or maintained for long periods); level 2 (investigate further-change may be required/should be introduced); level 3 (investigate further-change soon); and level 4 (change immediately).

In order to apply the RULA, researchers were authorized into the classroom during approximately 20 minutes for answering the questionnaire. This method evaluated orthostatism, chosen in view of the prolonged standing periods spent by professors during their occupational tasks. The remaining questionnaires were answered individually by the participants in the faculty lounge. We performed a descriptive analysis of the data using means, minimum and maximum values, and percentage distributions according to the characteristics of each variable. Possible correlations between RULA and MBI scores were analyzed using the Pearson correlation method. All analyses used SPSS version 20.0.

## RESULTS

This study had 17 participants, all active professors at the Serra Talhada campus of IF Sertão-PE; 9 of them (53%) were women and 8 (47%) were men, with a mean age of 35.30 years (SD, 6.28) (Table 1).

**Table 1:** Sociodemographic profiles of active faculty members of the Serra Talhada Campus of Instituto Federal do Sertão Pernambucano (IF Sertão-PE), 2019.

Variables	n (%)	Mean ± SD
Age		35,30 ± 6.28
Sex		
Female	9 (53.0)	
Male	8 (47.0)	
Daily workload (hours)		
6	1 (6.0)	
8	12 (71.0)	
More than 8	4 (23.0)	
Regular physical activity		
Yes	11 (65.0)	
No	6 (35.0)	

SD: standard deviation.

According to Table 1, 71% (n = 12) of the professors had a daily workload of 8 hours, and 65% (n = 11) had regular physical activity. Through the use of the NMQ (Table 2), we observed that 82% of the participants reported having felt some type of musculoskeletal symptom in the last 7 days, mostly in the lower back (52.9%) and lower limbs (29.4%).

**Table 2:** Prevalence of musculoskeletal symptoms by anatomical region in the last 7 days among active faculty members of the Serra Talhada campus of Instituto Federal do Sertão Pernambucano (IF Sertão-PE), 2019.

Anatomical region	n (%)
Neck	2 (11.8)
Shoulders	3 (17.6)
Arms	3 (17.6)
Elbows	0 (0.0)
Forearms	2 (11.8)
Wrists	2 (11.8)
Middle/upper back	3 (17.6)
Lower back	9 (52.9)
Lower limbs	5 (29.4)

The mean RULA score was 6.6 (Table 3), with action levels between 3 and 4, suggesting an imminent need for implementing posture changes since the workers are susceptible to developing occupational diseases. Table 3 also shows that the mean MBI score regarding EE was 37.94, and regarding DP it was 21.58. Both scores were considered high according to the MBI tool. The mean SR score was 25.29, considered representative of low self-realization.

**Table 3:** Rapid Upper Limb Assessment Overview (RULA) and Maslach Burnout Inventory (MBI) results presented by active faculty members of the Serra Talhada campus of Instituto Federal do Sertão Pernambucano (IF Sertão-PE), 2019.

Variable	Mean ± SD
RULA	6.6 ± 0.70
MBI	
EE	37.94 ± 6.53
DP	21.58 ± 3.27
SR	25.29 ± 5.34

SD: standard deviation; EE: emotional exhaustion; DP: depersonalization; SR: self-realization.

We could not identify statistically significant correlations between each of the MBI scales and the RULA results obtained through the questionnaires used in this study (p > 0.05) (Table 4).

**Table 4:** Pearson correlation analysis between the obtained variables of active faculty members of the Serra Talhada campus of Instituto Federal do Sertão Pernambucano (IF Sertão-PE), 2019.

Variables	Pearson correlation*
RULA and MBI	
EE	p = 0.498
DP	p = 0.161
SR	p = 0.224

RULA: Rapid Upper Limb Assessment; MBI: Maslach Burnout Inventory;EE: emotional exhaustion; DP: depersonalization; SR: self-realization

* p < 0.05

## DISCUSSION

Our results indicated predominantly female participants, which corroborated results published by Cardoso et al.^5^ and Mango et al.^16^ regarding education professionals. This could be explained by the process through which women entered the job market: many women chose professions linked to education considering it as an extension of the household labor, which included educating and caring for children^17^. Mean participant age was 35.30 years (SD, 6.28), slightly younger than those reported by Mango et al.^16^ and Lima Jr. & Silva^18^. Cardoso et al.^5^ observed an association between musculoskeletal pain and participants over 40 years old, attributing this effect to the natural wear and tear of the body, which leads to musculoskeletal discomfort.

Professors normally work long hours - most of the teachers studied by Silva & Guillo^19^ had daily workloads of 12 hours, which differed from our results (8 hours). Studies have shown that long working hours directly influence the health and lives of education professionals, and therefore excess work compromises mental and physical health, as well as family and social relationships^19,20^. Costa & Flausino^21^ reported that workers who had a weekly workload of 40 hours (approximately 8 hours/day) had more time for physical and leisure activities, in contrast with those that worked longer hours. In addition to having less free time for other activities, individuals working long hours are exposed to prolonged physical strain, which could justify the predominance of professors that had regular physical activity in our results, considering their weekly workloads. Moreira et al.^22^ also highlighted that most women had difficulties in maintaining regular physical activities due to the household labor performed after a regular workday.

The combination of occupational risk factors and lack of physical exercise can lead to the occurrence of RSI/WMSD; regular physical activity is of utmost importance in the prevention of various health problems and in promoting psychosocial benefits^23,24^. Our study verified a high prevalence of musculoskeletal symptoms according to the answers obtained in the NMQ: 82% of the participants reported musculoskeletal discomfort. These results are in line with those published by Mango et al.^16^, which showed that 91% of teachers presented some kind of musculoskeletal symptom; the authors have also associated this result to higher numbers of leaves and the need for occupational health care.

It is noteworthy that most reported symptoms were of the lower back and lower limbs, accounting for 52.9% and 29.4% of all reported symptoms, respectively. These results are in accordance with Lima Jr. & Silva^18^, who reported that professors presented musculoskeletal discomfort in these body areas. This high incidence could be attributed to poor posture and a high repetitiveness of the occupational activity, as well as lack of physical fitness for performing these activities^25^. Professors spend most of their workdays standing, which overloads and compresses the hip and knee joints, affecting their alignment. In the long term, the association of prolonged standing with physical inactivity and long working hours could lead to the wear and tear of the mentioned joints, thus contributing to musculoskeletal pain in the lower limbs^26^.

The analysis using the RULA tool verified that professors are exposed to postures that are considered risk factors and highlighted the importance of adopting preventive measures, such as alternating between sitting and standing postures and avoiding long periods of trunk flexion and repetitive movements^27^. The presence of an occupational physical therapist in companies or educational institutions could be considered a preventive measure, since this professional should evaluate the work environment and identify possible psychophysiological aspects that could trigger RSI/WMSD^28^.

Mean burn-out results were high in the EE and DP scales, in addition to low SR scores; these results are similar to those obtained by Carlotto & Palazzo^29^ and Batista et al.^30^, who associated these scores to work instability, long working hours, and occupational stress. The authors also noted that the reported signs and symptoms, although not necessarily prone to turn into severe mental disorders, are able to damage mental health and negatively affect the workers’ professional performance. Our results, when evaluated for correlations, did not present statistical significance, which could be explained by our small sample size and the heterogeneity of our results.

## CONCLUSIONS

According to our analysis, higher education professors reported high levels of musculoskeletal symptoms, mostly in the lower back and lower limbs. By using a RULA software tool, we highlighted the need for immediate changes in the postures adopted by these professionals when performing their work tasks. According to the MBI questionnaire, participants achieved high scores regarding the burn-out syndrome. Altogether, we recommend the implementation of an occupational physical therapist for performing ergonomic adaptations and elaborating protocols for workplace physical activities that aim to prevent musculoskeletal disorders and reduce occupational stress, thus preventing a high incidence of work-related diseases.
